# Transcriptomes of Arbuscular Mycorrhizal Fungi and Litchi Host Interaction after Tree Girdling

**DOI:** 10.3389/fmicb.2016.00408

**Published:** 2016-03-30

**Authors:** Bo Shu, Weicai Li, Liqin Liu, Yongzan Wei, Shengyou Shi

**Affiliations:** Key Laboratory of Tropical Fruit Biology, Ministry of Agriculture, South Subtropical Crops Research Institute, Chinese Academy of Tropical Agricultural ScienceZhanjiang, China

**Keywords:** litchi, carbohydrates, arbuscular mycorrhizal colonization, RNA-seq, transcripts

## Abstract

Trunk girdling can increase carbohydrate content above the girdling site and is an important strategy for inhibiting new shoot growth to promote flowering in cultivated litchi (*Litchi chinensis* Sonn.). However, girdling inhibits carbohydrate transport to the root in nearly all of the fruit development periods and consequently decreases root absorption. The mechanism through which carbohydrates regulate root development in arbuscular mycorrhiza (AM) remains largely unknown. Carbohydrate content, AM colonization, and transcriptome in the roots were analyzed to elucidate the interaction between host litchi and AM fungi when carbohydrate content decreases. Girdling decreased glucose, fructose, sucrose, quebrachitol, and starch contents in the litchi mycorrhizal roots, thereby reducing AM colonization. RNA-seq achieved approximately 60 million reads of each sample, with an average length of reads reaching 100 bp. Assembly of all the reads of the 30 samples produced 671,316 transcripts and 381,429 unigenes, with average lengths of 780 and 643 bp, respectively. Litchi (54,100 unigenes) and AM fungi unigenes (33,120 unigenes) were achieved through sequence annotation during decreased carbohydrate content. Analysis of differentially expressed genes (DEG) showed that flavonoids, alpha-linolenic acid, and linoleic acid are the main factors that regulate AM colonization in litchi. However, flavonoids may play a role in detecting the stage at which carbohydrate content decreases; alpha-linolenic acid or linoleic acid may affect AM formation under the adaptation process. Litchi trees stimulated the expression of defense-related genes and downregulated symbiosis signal-transduction genes to inhibit new AM colonization. Moreover, transcription factors of the AP2, ERF, Myb, WRKY, bHLH families, and lectin genes altered maintenance of litchi mycorrhizal roots in the post-symbiotic stage for carbohydrate starvation. Similar to those of the litchi host, the E3 ubiquitin ligase complex SCF subunit scon-3 and polyubiquitin of AM fungi were upregulated at the perceived stages. This occurrence suggested that ubiquitination plays an important role in perceiving carbohydrate decrease in AM fungi. The transcription of cytochrome b-245 and leucine-rich repeat was detected in the DEG database, implying that the transcripts were involved in AM fungal adaptation under carbohydrate starvation. The transcriptome data might suggest novel functions of unigenes in carbohydrate shortage of mycorrhizal roots.

## Introduction

Arbuscular mycorrhiza represents widespread mutualistic association between soil-borne fungi of the Phylum Glomeromycota and most land plants. AM fungi play a key role in the life of host plants; the fungi supply mineral nutrients to the roots (biofertilizers), influence plant development (bioregulators), and enable mycorrhizal plants to overcome biotic and abiotic stresses (bioprotectors; Parniske, 2008; Smith and Read, 2008). The host plant provides carbohydrates to obligate biotrophic fungi to enable the fungi to complete their life cycle. Plants could allocate up to 30% of their photosynthetic carbohydrates to AM fungi, which in return, provides up to 80% of plant phosphate and nitrogen. Moreover, the two partners guarantee a “fair trade” of mineral nutrients against carbohydrates (Kiers et al., 2011; Fellbaum et al., 2012). Although carbohydrates provided by host plants play important roles in the life cycle of AM fungi, knowledge on processes involved in the host carbohydrate regulation for AM fungi colonization remains limited.

Litchi (*Litchi chinensis* Sonn.), which belongs to the Sapindaceae family, is an important woody mycorrhizal fruit tree in southern China. The area for cultivation of litchi reached 553,000 ha in 2015. The main cultivars of litchi (*L. chinensis* Sonn. Feizixiao and *L. chinensis* Sonn. Guiwei) normally flower in March and mature in June in the Guangdong province of China. Trunk girdling increases carbohydrate content above the girdling site and decreases the uptake of water and mineral nutrition; this strategy is important to inhibit new shoot growth and promote flowering in cultivated litchi (Yuan and Huang, 1993; Huang et al., 2003). Trunk girdling is generally performed in the second half of November before the period of flower-bud differentiation and wound healing in half a year. However, girdling adversely affects carbohydrate transport to the root of litchi, thereby restricting litchi mycorrhizal root development. This technique also reduces mycorrhizal root absorption, consequently decreasing the quality of fruit or inducing fruit drop during fruit development. Hence, the coping strategy of litchi mycorrhizal root under carbohydrate starvation must be elucidated to improve culture techniques.

Carbohydrates in plant tissues are diverse throughout evolution; the different kinds of carbohydrates include glucose, fructose, sucrose, sorbitol, and starch in the roots of the apple tree (Tromp, 1983) as well as glucose, fructose, sucrose, quebrachitol, and starch in the root of the litchi tree (Wang et al., 2013). All types of carbohydrates starvation may affect the pre- and post-symbiotic interactions of the AM fungi and the host plant. To date, several molecular signals are identified and used to recognize AM fungi and host plant in the pre-symbiotic period. Flavonols, strigolactones, and hydroxy fatty acids from the host plant and lipochito-oligosaccharides, short-chain chitin oligomers, and steroids from the AM fungi are dialog components (Bécard et al., 1992; Besserer et al., 2008; Maillet et al., 2011; Nagahashi and Douds, 2011; Genre et al., 2013; Bucher et al., 2014; Sun et al., 2015). Molecules and related genes in the post-symbiotic stage are more complex than those in the pre-symbiotic period. Genes, which are related to phytohormone biosynthesis and signal transduction, transport systems of nutrient and carbohydrate, transcription factor, and other processes, all participate in the post-symbiotic stages (De Hoff et al., 2009; Hogekamp et al., 2011; Handa et al., 2015). To analyze the influence of carbohydrate starvation on the litchi mycorrhizal root, this study focused on mycorrhizal root carbohydrates (glucose, fructose, sucrose, quebrachitol, and starch) to determine the degree of carbohydrate decrease and measure AM colonization rate to clarify whether carbohydrates reduce AM colonization. Based on the above analysis, this study also tested the transcriptome data to screen for interesting transcripts involved in the interaction between AM fungi and host litchi under carbohydrate starvation.

## Materials and Methods

### Plant Material and Cultivation Conditions

Six litchi trees with middle growth vigor were selected from 200 trees (cultivar Feizixiao, grafted on 12-year-old Huaizhi rootstock) and cultivated in the experimental orchard of the South Subtropical Crops Research Institute, Zhanjiang, China (mineral nutrition is shown in Supplementary Table 1). The trees received standard horticultural practices (such as weeding and irrigating) as well as disease and insect control. Three of the six trees were girdled in November (The girdling picture was shown in **Supplementary Figure S1**), and the remaining trees were used as control. Root (diameter < 1.5 mm) was sampled to 20–30 cm soil thickness, where fibrous roots were overdispersed, from the four directions of each tree; duplicate samples were obtained from each tree. The root samples were immediately obtained after girdling treatment (0 day, 0 D) as well as 1 week, 2 weeks, 1 month, and 2 months (1 W, 2 W, 1 M, and 2 M, respectively) thereafter. Thirty samples were acquired in the experiment. The samples were washed with sterile water, wiped dry with gauze at harvest, frozen in liquid nitrogen, and then immediately stored at -80°C.

### Root Mycorrhizal Colonization

A fraction of the fresh roots was fixed in formalin/acetic acid/ethanol (FAA, 13:5:200 [v/v/v]) for 24 h to determine the degree of AM colonization. The roots were cleared in 10% (w/v) KOH at 99°C for 1.5 h and stained with 0.05% (w/v) trypan blue in lactophenol by using methods previously described by Phillips and Hayman (1970). The structures of AM fungi were examined under a compound light microscope (Olympus-BH-2). Fungal colonization was estimated using magnified intersection method (McGonigle et al., 1990). Total AM and arbuscule colonization rate were quantified by examining 200 intersections for each sample.

### Measurements of Soluble Sugars and Starch

Soluble sugars were measured using HPLC analyses. Determinations were performed as described by Wang et al. (2013) and Wang T.D. et al. (2014). About 1 g of the roots were homogenized, extracted three times with 85% (v/v) ethanol (3 mL each), and then centrifuged at 6000 × *g* for 10 min. The pooled supernatant was rotary evaporated to dryness and resuspended in 4 mL of distilled water. The precipitate was then analyzed for starch content. Sugars were detected by Agilent 1200 HPLC system (Agilent Technologies, Waldbronn, Germany) equipped with a refractive index detector and a transgenomic CARB Sep Coregel 87C column (CHO-99-5860). The ultra-pure water was the mobile phase at a flow rate of 0.8 mL/min. The sugars were identified by comparing their retention times with those of authentic standards. The concentrations of individual sugars were quantified using peak areas and calibration curves derived from the standards.

The precipitates were combined with 2 mL of water, and the mixture was gelatinized for 15 min in boiled water. The mixture was cooled to 20°C and then added with 2 mL of 9.2 M perchloric acid. The mixture was stirred for 15 min, combined with 4 mL of water, and centrifuged at 3500 × *g* for 10 min. The supernatant was transferred to a 50 mL volumetric flask. The precipitate was combined with 2 mL of 4.6 M perchloric acid, and the resulting mixture was stirred for 15 min. The mixture was added with 5 mL of water and centrifuged at 3500 × *g* for 10 min. The supernatant was then transferred to the same 50 mL volumetric flask. The precipitate was washed twice with 5 mL of water, and the water was also transferred to a 50 mL volumetric flask. The solution in the 50 mL volumetric flask was analyzed for starch content according to Wang T.D. et al. (2014).

### Total RNA Extraction and RNA Sequencing

Total RNA was isolated from the mycorrhizal root of litchi by using TRIzol reagent (Invitrogen, USA) and treated with DNase I to eliminate genomic DNA contamination. Three biological replicates were prepared for each treatment, and RNA of 30 samples was obtained. RNA quality was determined using Agilent 2100 Bioanalyzer. After the total RNA extraction and DNase I treatment, magnetic beads with Oligo (dT) were used to isolate mRNA. The isolated mRNA was mixed with the fragmentation buffer and then fragmented into short fragments. cDNA was synthesized using the mRNA fragments as templates. Short fragments were purified and resolved with elution buffer for end reparation and addition of single nucleotide A (adenine). The short fragments were connected with adapters. Suitable fragments were selected for PCR amplification as templates. During the quality control, Agilent 2100 Bioanalyzer and ABI StepOnePlus Real-Time PCR System were used for quantification and qualification of the sample library. Finally, 30 libraries were sequenced using the Illumina HiSeq 2000 system.

### Transcript Assembly and Functional Annotation

Prior to bioinformatic analysis, the raw sequences were filtered to remove reads that contained only the adaptor sequences, those with more than 5% unknown nucleotides, and the low-quality reads with more than 20% bases having a quality value ≤ 10. Because litchi genome has not been published and multiplex types of AM fungi which might show sequence difference from *Rhizophagus irregularis* are existed in litchi mycorrhizal root, *de novo* assembly was performed by the Beijing Genomics Institute using the short-read assembly program for both litchi and AM fungi by using the Trinity software (version 2.0.6; Grabherr et al., 2011). The Blast software (version 2.2.30+) with evalue 1e-3 -num_alignments 1 was used for separating the transcript of litchi and AM fungi by against the genome (*R. irregularis* DAOM 197198, Gloin1^1^) and nr database^2^.

TransDecoder software (version r20140704) was employed to predict the transcript open reading frame with -m50 setting. Functional annotation of the unigenes was performed using the nr database, the Swiss-Prot protein database^3^, the KEGG database^4^, uniref 90^5^, and Gloin1 by using BLASTx with an *E*-value < 10^-5^ by Trinotate software (version 20140708). When a unigene did not align to any of the above databases, Hmmscan (HMMER) software (version 3.1) was used for annotation through functional domain prediction (Grabherr et al., 2011).

### Read Mapping and Quantification of Gene Expression

Reads containing adaptors, reads with more than 10% unknown nucleotides, and low-quality reads with more than 50% bases with a quality value ≤ 5 were removed to obtain uncontaminated sequences. The uncontaminated sequences from each sample were mapped to the assembly transcripts by using Bowtie software (version: 1.0.1; parameters: mismatch = 2). The files of the bam format were achieved and used to calculate for the number of reads mapped on the transcript by RSEM software (version: v1.2.17). The number of mapped and filtered reads for each unigene was calculated to obtain the corresponding FPKM values (Li and Dewey, 2011). DEGs between two samples were determined using the FDR threshold of <0.001, an absolute log twofold change value of >1.0, and a *P*-value of <0.01 by Edger (version: 3.10.2). The GO and KEGG pathway analyses of DEGs were accomplished using GOseq software (version: 3.0) and KOBAS software (version: 2.0; Young et al., 2010; Xie et al., 2011). The heat maps of the selected DEGs from litchi and AM fungi were constructed using mev software (v4.9.0). The flow chart for RNA-seq analysis was shown in **Supplementary Figure S2**.

### Statistical Analysis

Experimental data were statistically analyzed using ANOVA through SAS 8.1 software (SAS Institute, Cary, NC, USA). The probabilities of significance were used to determine significance among the treatments, and the least-significant difference (*p* < 0.05) was used to compare the data.

## Results

### Carbohydrates

**FIGURE 1:**
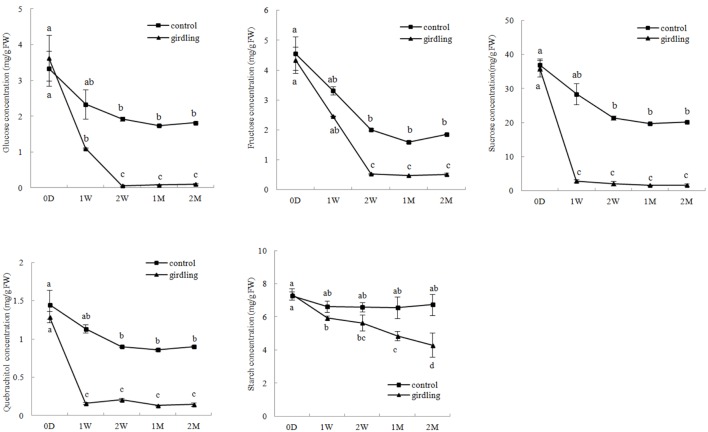
**Carbohydrate variation in the litchi mycorrhizal roots after girdling treatment.** The C represents control, and the G represents girdling; 0 D, 1 W, 2 W, 1 M, 2 M represent the time after treatment (0 day, 1 week, 2 weeks, 1 month, and 2 months).

The HPLC results showed that sucrose concentration (1.59–46.96 mg/g⋅FW) was higher than the other carbohydrate contents in the litchi mycorrhizal roots. Variation in glucose, fructose, sucrose, and quebrachitol contents in the control and girdled mycorrhizal roots exhibited the same pattern. This finding revealed that continuous decrease began at 0 D to 2 W after girdling and then remained stable from 2 W to 2 M. The concentrations of glucose, fructose, sucrose, and quebrachitol in the control were significantly higher than those in the girdled group from 1 W to 2 M. In contrast to other carbohydrates, starch concentration in the girdling treatment continuously decreased and significantly differed from 1 to 2 M compared with that of the control (**Figure 1**).

### Root Mycorrhizal Colonization

**FIGURE 2:**
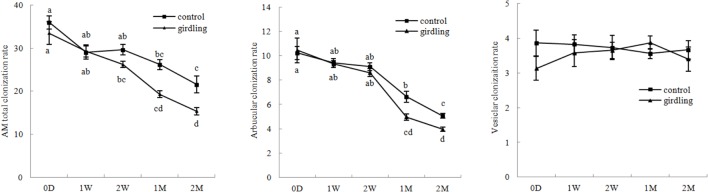
**AM colonization variation in the litchi mycorrhizal roots after girdling treatment.** The C represents control, and the G represents girdling; 0 D, 1 W, 2 W, 1 M, 2 M represent the time after treatment (0 day, 1 week, 2 weeks, 1 month, and 2 months).

The rates of total, arbuscular, and vesicular colonization rates of litchi were 15.33–36.00%, 3.97–10.43%, and 3.10–3.90%, respectively, under field conditions. Variation in the total and arbuscular colonization rates continuously decreased from 0 D to 2 M. The total and arbuscular colonization rates were significantly higher in the control than those in the girdling treatment at 1 and 2 M. Vesicular colonization was not significantly different from total and arbuscular colonizations after girdling (**Figure 2**).

### Sequence Assembly and Annotation

Thirty samples were sequenced using the Illumina genome analyzer Hiseq 2000. After quality checking and data cleaning, approximately 60 million reads of each sample were obtained, with average lengths reaching 100 bp and GC content of 44–46% (Supplementary Table 2). Assembly of all the reads of the 30 samples generated 671,316 transcripts and 381,429 unigenes, with average lengths of 780 and 643 bp, respectively. A total of 308824 unigenes were annotated among all unigenes (**Table 1**). These transcriptome data were submitted to sequence read archive of NCBI with the NO. SRX1518711.

**Table 1:** Summary of read numbers based on the RNA-Seq data from the mycorrhizal roots of litchi after girdling treatment.

Item	Unigenes	Transcripts
Total number	381,429	671,316
N20	2,113	2,507
Median length	390	469
Average length	643	780
Total length	245,635,054	524,004,633

**Item**	**Number of genes**	**Percentage**

All	381429	100.00%
Annotated	308824	80.97%
Blast hit	253077	66.35%
Pfam	138101	36.21%
Gene ontology	157447	41.28%
Eggnog	82759	21.70%
SignalP	15636	4.10%
TmHMM	43358	11.37%

**FIGURE 3:**
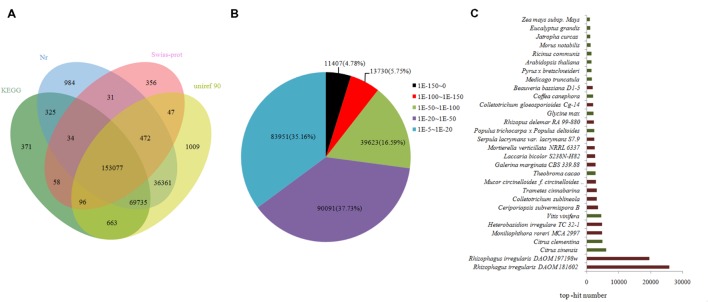
**Outcome of homology search of unigenes against the nr, KEGG, Swiss-prot, and uniref 90 databases. (A)** Venn diagram of unigene numbers annotated by BLASTx with an *E*-value threshold of 10^-5^ against protein databases. The numbers in the circles indicate unigenes numbers annotated by single or multiple databases. **(B)** *E*-value distribution of the top BLAST hits for each unique sequence. **(C)** Species distribution of the top BLAST hits for all homologous sequences. Those indicated in brown denote AM fungi, and those in green correspond to litchi in **(C)**.

Approximately 262,619 unique sequences were annotated through BLASTx (cut-off *E*-value10^-5^) search of four public databases, namely, nr, Swiss-Prot protein, KEGG, and uniref 90. Of the sequences, 260,019 unique sequences were annotated with reference to the nr database, whereas 2,600 unigenes were annotated using the other databases (**Figure 3A**). The results of the annotation indicated that 9.66% (25,137) of the annotated sequences exhibited “very strong homology” (*E*-value < 10^-100^), 15.08% (39,623) exhibited “strong homology” (10^-100^ < *E*-value < 10^-50^), and 70.26% (174,042) displayed “homology” (10^-50^< *E*-value < 10^-5^) to the available sequences (**Figure 3B**). The unique sequences of AM fungi achieved top matches to sequences from *R. irregularis* DAOM181602 and *R. irregularis* DAOM197198w. The unique sequences of litchi exhibited top matches to sequences from *Citrus sinensis* and *Citrus clementina* (**Figure 3C**).

**FIGURE 4:**
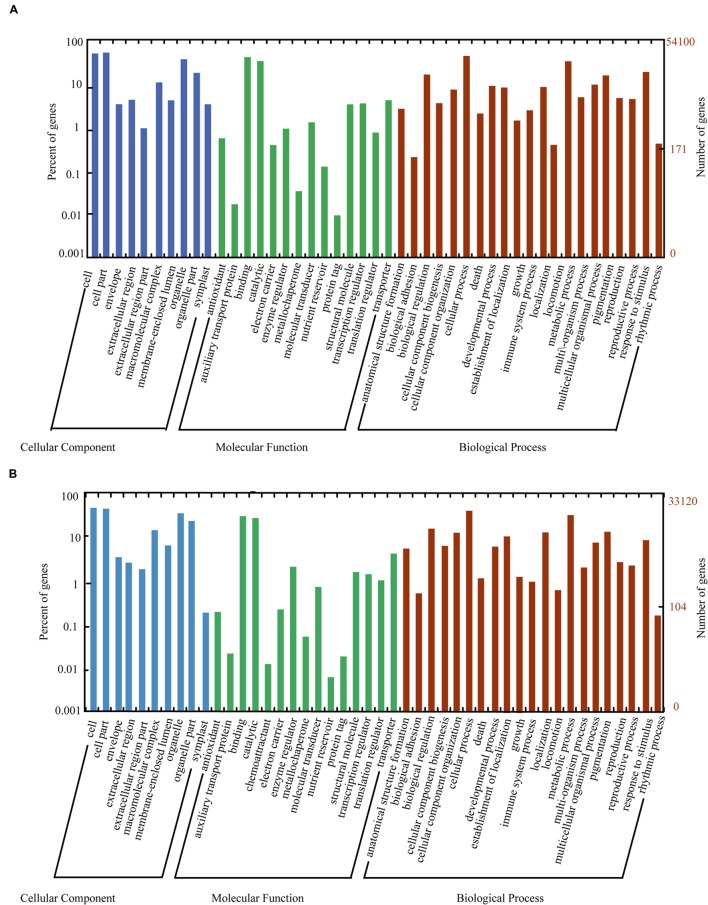
**Histogram of GO classifications for litchi **(A)** and AM fungi **(B)** transcripts in mycorrhizal roots.** The unigenes corresponded to three main categories: “biological process,” “cellular component,” and “molecular function.” The left- and right-hand *y*-axes indicate the percentage and number of annotated unigenes, respectively.

The nr, GO, KEGG, and uniref 90 databases were used to classify the functions of the predicted unigenes. The unigenes of litchi (54,100 unigenes) and AM fungi (33,120 unigenes) were classified into three main categories as follows: “cellular component,” “molecular function,” and “biological process” (**Figure 4**). Meanwhile, the host litchi and AM fungi obtained numerous unigenes annotated as “cell and organelle” in the “cellular component” category; “binding” and “catalytic activities” in the “molecular function” category; and “metabolic process,” “cellular process,” “response to stimulus,” and “biological regulation” as four subcategories in the “biological process” category. However, the rates of the same subcategories differed between litchi and AM fungi. Within the “cellular component,” the subcategory “extracellular region part” attained a rate of less than 1% in litchi but more than 1% in fungi. The subcategory “symplost” showed more than 1% rate in litchi but less than 1% in AM fungi. Within the “molecular function” category, the subcategory of “chemoattractant” resulted in 0.01% rate in fungi but was not found in the litchi component. The subcategory of “nutrition reviser” was more than 0.01% in litchi and less than 0.01% in AM fungi. Within “biological process,” the subcategory of “immune system process” in litchi unigenes was more than 1% but less than 1% in fungi (**Figure 4**).

### Differential Gene Expression Analysis

The DEGs after girdling were assessed by pair-wise comparisons of all time points with the expression fold (log 2 Ratio ≥ 1) and FDR ≤ 10^-3^ as the thresholds in litchi and AM fungi (**Table 2**). The number of DEGs increased with prolonged girdling. Thirty-four DEGs of litchi (34 upregulated and 0 upregulated) and only one DEG of AM fungi (1 upregulated and 0 downregulated) were detected between 0 D and 1 W. Moreover, 442 DEGs of litchi (286 upregulated and 156 downregulated) and 742 DEGs of AM fungi (742 upregulated and 0 downregulated) were identified at 0 D and 2 M after pair-wise comparison (**Table 2**).

**Table 2:** The quantity of DEGs in litchi mycorrhizal roots after girdling treatment.

	0 Day		1 Week		2 Week		1 Month		2 Month	
	Up	Down	Up	Down	Up	Down	Up	Down	Up	Down
0 D	0/0	0/0	34/1	0/0	99/41	108/0	54/190	106/0	286/742	156/0
1 W			0/0	0/0	10/5	27/1	2/4	47/0	135/244	23/1
2 W					0/0	0/0	6/5	12/0	507/79	91/4
1 M							0/0	0/0	218/19	30/2
2 M									0/0	0/0

*The constitution of each value was ‘DEG number of litchi/DEG number of AM fungi.’*

### KEGG Pathway Enrichment Analysis of Differentially Expressed Genes

The main biological process and related unigenes were screened from host litchi and AM fungi during carbohydrate starvation. Most upregulated DEGs at 0 D versus those at 1 W as well as at 0 D versus those at 2 W were mapped to carbohydrate metabolism, organismal systems (plant–pathogen interaction), and amino-acid metabolism for litchi. In particular, the upregulated DEGs were mapped to carbohydrate metabolism pathways, such as pyruvate metabolism (ko00620), amino sugar and nucleotide sugar metabolism (ko00520), and starch and sucrose metabolism (ko00500). For organismal systems, the upregulated DEGs were mapped to the plant–pathogen interactions (ko04626). The amino-acid metabolism, such as phenylalanine metabolism (ko00360) as well as valine, leucine, and isoleucine biosynthesis (ko00290) were upregulated (Supplementary Table 3). The downregulated DEGs at 0 D versus those at 1 W and at 0 D versus those at 2 W were clustered into biosynthesis of other secondary metabolites, carbohydrate metabolism, and endocrine system. Most downregulated transcripts were then mapped to the biosynthesis of other secondary metabolites in flavonoid biosynthesis (ko00941), flavone and flavonol biosynthesis (ko00944), and phenylpropanoid biosynthesis (ko00940); carbohydrate metabolism, including starch and sucrose metabolism (ko00500); and amino sugar and nucleotide sugar metabolism (ko00520; Supplementary Table 3). In the adaptation process, most upregulated DEGs at 0 D versus those at 1 M and at 0 D versus those at 2 M in litchi were clustered into lipid metabolism and biosynthesis of other secondary metabolites. The upregulated transcripts mapped to lipid metabolism were grouped under alpha-linolenic acid metabolism (ko00592), linoleic acid metabolism (ko00591), and sphingolipid metabolism (ko00600). The biosynthesis of other secondary metabolites, including phenylpropanoid (ko00940); stilbenoid, diarylheptanoid, gingerol (ko00945); and flavonoids, (ko00941) were also upregulated. The downregulated DEGs were mapped to several clusters under carbohydrate metabolism and immunity. The transcripts mapped to carbohydrate metabolism were grouped under starch and sucrose metabolism (ko00500), amino sugar and nucleotide sugar metabolism (ko00520), and pentose phosphate pathway (ko00030). Meanwhile, the transcripts mapped to the immune system were grouped under antigen processing and presentation (ko04612) and the NOD-like receptor signaling pathway (ko04621; Supplementary Table 3).

The upregulated DEGs at 0 D versus 1 W and at 0 D versus 2 W in AM fungi were mapped to several pathways under genetic information processing and carbohydrate metabolism. Genetic information processing included protein processing in the endoplasmic reticulum (ko04141) and ribosome (ko03010), as well as ubiquitin-mediated proteolysis (ko00290). Under carbohydrate metabolism, glyoxylate and dicarboxylate metabolism (ko00630), citrate cycle (tricarboxylic acid cycle; ko00020), and pyruvate metabolism (ko00620) were noted. The upregulated DEGs at 0 D versus those at 1 M and at 0 D versus those at 2 M in the AM fungi were mapped to genetic information processing, amino acid metabolism, lipid metabolism, and signal transduction. The genetic information processing included RNA transport (ko03013), RNA degradation (ko03018), mismatch repair (ko03430), protein processing in the endoplasmic reticulum (ko04141), and ubiquitin-mediated proteolysis (ko04120). Meanwhile, amino acid metabolism included alanine, aspartate, and glutamate metabolism (ko00250) as well as arginine and proline metabolism (ko00330). For lipid metabolism, fatty-acid biosynthesis (ko00061), alpha-linolenic acid metabolism (ko00592), and biosynthesis of unsaturated fatty acids (ko01040) were found. For signal transduction, MAPK (ko04011), Ras (ko04014), and sphingolipid signaling pathways (ko04071) were noted. The downregulated DEGs of the AM fungi were few; these DEGs were noted at 1 W versus those at 2 W (TR92985| c2_g2, putative cruciform DNA binding protein), at 1 W versus those at 2 M (TR92985| c2_g2, putative cruciform DNA binding protein), at 2 W versus those at 2 M (TR5997| c0_g2, putative protein far1-related sequence 10; TR155121| c0_g1, putative pyruvate decarboxylase; TR146357| c0_g2 and TR95299| c0_g2 uncharacterized protein), and at 1 M versus those at 2 M (TR92985| c2_g2, putative cruciform DNA binding protein; TR95299| c0_g2, uncharacterized protein). Only TR155121| c0_g1 was mapped to the glycolysis/gluconeogenesis pathway (ko00010; Supplementary Table 3).

### Genes Including Ubiquitination, Transcription Factor, and with Repeated Domain

**FIGURE 5:**
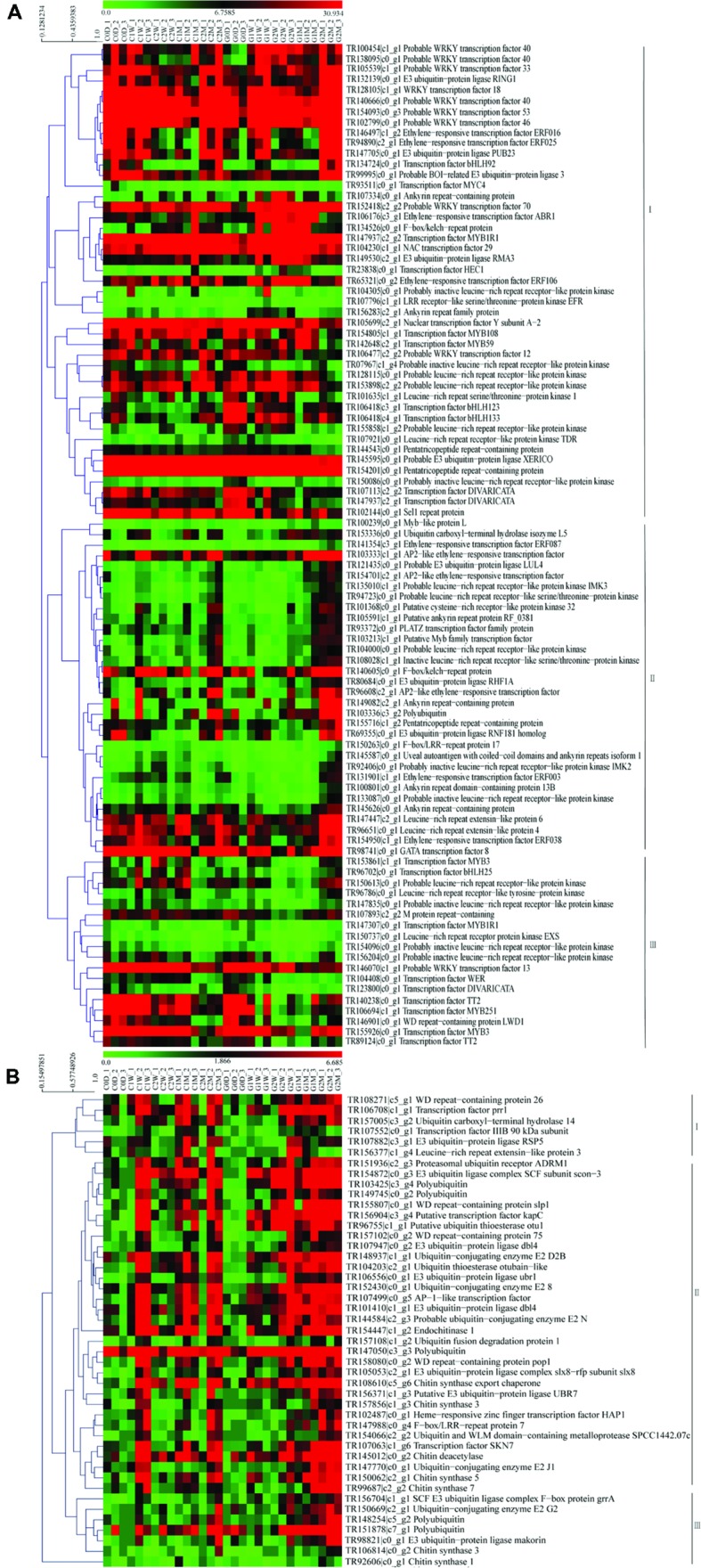
**Heat map of the genes including ubiquitination, transcription factor and repeated domain expression for litchi **(A)**; heat map of the genes including ubiquitination, transcription factor, repeated domain expression, and chitin synthesis for AM fungi **(B)**.** The C represents control and the G represents girdling; 0 D, 1 W, 2 W, 1 M, 2 M represent the time after treatment (0 day, 1 week, 2 weeks, 1 month, and 2 months). The heat map was constructed based on the FPKM values which showed on the top of the figures by the software mev (v4.9.0).

Ubiquitination-related unigenes, transcription factor, and unigenes with repeated domains in the host litchi were selected to construct heat map A. Ubiquitination-related unigenes, transcription factor, unigenes with repeated domains, and unigenes related to chitin synthesis in the AM fungi were used to construct heat map B. The heat map of the host litchi revealed that all selected unigenes were divested into three main subclusters. Unigenes in subcluster I were upregulated by girdling on 2 W and 1 M; unigenes in subcluster II were induced by girdling on 2 M; and unigenes in subcluster III were downregulated by girdling from 0 D to 2 M (**Figure 5A**). The heat map of AM fungi showed that all of the selected unigenes were clustered into three main subclusters. Unigenes in subcluster I were downregulated by girdling from 0 D to 1 W but were upregulated from 2 W to 2 M. Unigenes in subcluster II were downregulated by girdling on 1 W but upregulated from 2 W to 2 M. Furthermore, unigenes in subcluster III were upregulated by girdling on 2 M (**Figure 5B**).

### Clustering Results of Time-Course Data from RNA-Seq by STEM Analysis

**FIGURE 6:**
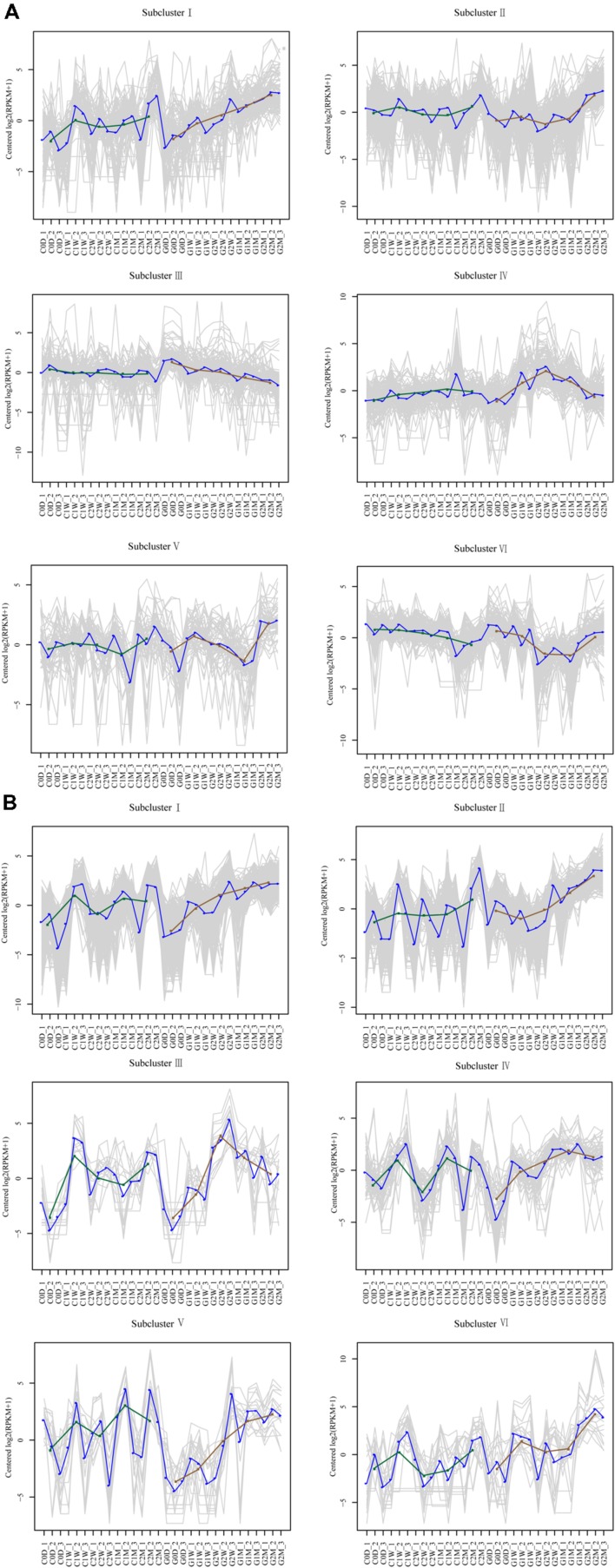
**Clustering results of time-course data from RNA-seq by short time-series expression miner analysis for litchi **(A)** and AM fungi **(B)**.** Each box corresponds to one of the model temporal expression profiles, and the unigene belonging to each corresponding subcluster is shown in Supplementary Table 4. The C represents control, and the G represents girdling; 0 D, 1 W, 2 W, 1 M, 2 M represent the time after treatment (0 day, 1 week, 2 weeks, 1 month, and 2 months).

DEGs with similar expression patterns were clustered into six distinct subclusters for AM fungi and host litchi, and DEGs grouped in the same subcluster may be functionally correlated. Most DEGs in the host litchi were clustered into subclusters I and II, which were upregulated in the girdling treatment (**Figure 6A**). The expression levels of DEGs in subcluster III were stable in the control but downregulated in the girdling treatment. The expression levels of DEGs in subcluster IV were stable in the control but induced in girdling at 2 W. The expression of DEGs in subcluster V were similar to those in the control and girdling treatment from 0 D to 1 M, but the expression levels in the latter were strong upregulated at 2 M. The expression levels of DEGs in subcluster VI of the control were downregulated from 0 D to 2 M but were induced by girdling at 2 M (**Figure 6A**). Similar to those in the host litchi, the numbers of genes assigned among the six clusters in the AM fungi were statistically significant (**Figure 6B**). The DEGs of subcluster I were upregulated from 1 W in the girdling treatment, and those of subcluster II were upregulated from 2 W. The expression patterns of DEGs in subcluster III were upregulated after 2 W of girdling and then downregulated from 2 W to 2 M. The expression patterns of DEGs in subcluster IV were also upregulated after 2 W of girdling and maintained at high levels from 2 W to 2 M. The expression levels of DEGs in subcluster V were lower than those in the control from 0 D to 2 W but were induced by girdling from 2 W to 2 M. The expression patterns of DEGs in subcluster VI showed the same patterns in the control and girdling groups, but the expression levels in the latter were higher than those in the former (**Figure 6B**; Unigenes of each subcluster were displayed in Supplementary Table 4).

## Discussion

RNA-seq is a technique used to detect low-expressing reads (Garber et al., 2011; Wang C. et al., 2014) and identify novel transcripts in AM with DEG screening (Fiorilli et al., 2015; Handa et al., 2015). In the present experiment, transcripts obtained from each sample included litchi- and fungi-derived transcript as well as transcripts that originated from other microorganisms. The *R. irregularis* genome, genomes of model plants, and those stored in other databases were used to distinguish the transcripts, whether they were derived from litchi, AM fungi, or other organisms, through BlastP prediction (Tisserant et al., 2013). RNA-seq analysis showed approximately 60 million reads for each sample, with average read length of 100 bp. The assembly of all reads of the 30 samples produced 671,316 transcripts and 381,429 unigenes, with average lengths of 780 and 643 bp, respectively. BlastX analysis revealed that 10.85% (25,920) of the unigenes were top matched to sequences from AM fungus *R. irregularis* DAOM 181602, and 8.22% (19,627) were matched to sequences from AM fungus *R. irregularis* DAOM 197198. The unigenes of litchi (54,100 unigenes) and AM fungi (33,120 unigenes) were identified by sequence annotation during the last period of carbohydrate decrease (**Figure 4**). More than 1,300 DEGs from host litchi and 1,000 DEGs from AM fungi were noted for the corresponding carbohydrate decrease (**Supplementary Figure S2**; Supplementary Table 4). The researches on transcriptome-related AM symbiosis were obtained by different methods, such as SuperSAGE analysis, Genechip and RNA-seq. SuperSAGE analysis showed 32,808 SuperSAGE Tag sequences matched to *Nicotiana attenuata* transcriptome database and 3,698 Tag sequences matched to *R. irregularis.* Genechip was used for DEGs screening in transcriptome of *Medicago truncatula* and *Solanum lycopersicum* mycorrhizal root (Gaude et al., 2012; Ruzicka et al., 2012; Bonneau et al., 2013; Hogekamp and Küster, 2013). It identified 512 DEGs between AM colonized and non-colonized cells in *M. truncatula* root (Gaude et al., 2012); 174 DEGs between mycorrhizal wild-type and non-mycorrhizal *rmc* roots irrespective of the N treatment of *Solanum lycopersicum* under field condition (Ruzicka et al., 2012). RNA-seq analysis identified 2,210 fungal and tomato sequence assemblies from mycorrhizal roots through comparing the wild-type and *rmc* root samples under field condition (Ruzicka et al., 2013); 3,641 genes differentially expressed during AM development in *Lotus japonicus* and approximately 80% of which were upregulated (Handa et al., 2015); 3,949 DEGs between mycorrhizal roots of large lateral roots and fine lateral roots in *Oryza sativa* (Fiorilli et al., 2015). The numbers of annotated sequences of host litchi and AM fungi in present study compared with those annotated sequences and DEGs in the biological process of AM fungi and other host plants as well as DEGs involved in carbohydrate starvation suggested that RNA-seq is an effective method for identifying transcript variation in litchi mycorrhizal roots under decreased carbohydrate conditions (**Table 2**).

Previous research suggested that plants can detect, discriminate, and reward the optimal fungal partners with high carbohydrate contents. Moreover, the fungal partners encourage cooperation by increasing nutrient transfer to the hosts to provide high amounts of carbohydrates (Kiers et al., 2011; Fellbaum et al., 2012). Mineral nutrients against carbohydrates between partners were characteristic of both sucrose starvation and hexose-enhanced conditions. Sucrose is the main sugar for transport in *M. truncatula*; the lines displayed up to 10-fold reduction in the expression levels of the *M. truncatula* sucrose synthase gene (*MtSucS1*) in the roots (Baier et al., 2010). The lines exhibited decreased numbers of internal hyphae, vesicles, and arbuscules, which caused an overall stunted aboveground growth under inorganic phosphorus limitations (Baier et al., 2010). Hexoses, especially glucose, are the main carbohydrates for mineral nutrition from AM fungi (Helber et al., 2011). Invertase derived from overexpressing yeast (*Saccharomyces cerevisiae*) in tobacco (*N. tabacum*) *alc::cwINV* increased the hexose concentration in the root. However, the colonization of *Glomus intraradices* and the level of fungus-specific palmitvaccenic acid, which indicate fungal carbohydrate supply, or plant phosphate content, did not increase (Schaarschmidt et al., 2007). These results implied that sufficient carbohydrates are available in mycorrhizal roots under normal conditions, but carbohydrate starvation inhibited AM development. As predicted, girdling decreased starch concentration as well as monosaccharide, disaccharide, and sugar alcohol contents after the treatment. AM colonization rate matched carbohydrate variation, which continuously decreased after the girdling treatment from 2 W to 2 M (**Figures 1** and **2**). Besides decreased carbohydrate content and AM colonization, DEGs generated by girdling also suggested that both the litchi root and AM fungi underwent carbohydrate starvation. Biological processes related to carbohydrate metabolism varied after the girdling treatment; these processes include glyoxylate and dicarboxylate metabolism (ko00630), citrate cycle (ko00020), and pentose phosphate pathway (ko00030) in AM fungi; as well as starch and sucrose metabolism (ko00500) and amino sugar and nucleotide sugar metabolism (ko00520) in litchi (Supplementary Table 3). The results, including carbohydrate content, AM colonization, and pathways in both host plant and AM fungi, suggested that girdling regulated litchi mycorrhizal root development by decreasing carbohydrate loss.

Carbohydrate shortage in symbiotic roots lowers AM fungi colonization and decreases the proportion of functional arbuscules (Hayman, 1974; Vierheilig et al., 2002). Girdling inhibits mycorrhizal development in both symbiotic partners. We speculate that upregulated DEGs are required for the detection and adaptation of carbohydrate stress. By contrast, downregulated DEGs in symbiotic partners may be related to carbohydrate exchange under normal conditions. Flavonoids are not strictly required for all combinations of host plant and AM fungi recognition (Bécard et al., 1995). However, the downregulated DEGs at 0 D versus 1 W and at 0 D versus 2 W in host litchi were clustered into flavonoid, flavone, and flavonol biosynthesis as systemic processes. The evidence may suggest that flavonoid was the main factor that regulated AM formation in litchi. Compared with the downregulated DEGs, most of the upregulated DEGs were clustered into stress-inducible and resistance-related pathways, which included the NADP-dependent malic enzyme gene, endochitinase PR4, the basic form of the pathogenesis-related protein 1 LRR receptor-like serine/threonine-protein kinase EFR, cyclic nucleotide-gated ion channel 1, lignin-forming anionic peroxidase, and peroxidase 72 (Gallou et al., 2012; Tromas et al., 2012; Miyata et al., 2014). These data implied that the host litchi stimulated defense-related genes in response to fungal disease when its seceded carbohydrates decreased. In the adaptation process, the downregulated DEGs were mapped to several clusters, including antigen processing and presentation (heat shock protein 82; Heat shock 70 kDa protein; putative heat shock protein HSP 90-beta-3; nuclear transcription factor Y subunit A-2), plant hormone signal transduction (transcription factor MYC4; probable protein phosphatase 2C 24; regulatory protein NPR5), NOD-like receptor signaling pathway (heat shock protein 82; putative heat shock protein HSP 90-beta-3), and MAPK signaling pathway (heat shock 70 kDa protein). The data showed that when carbohydrate shortage, the litchi might downregulate the symbiosis signal-transduction genes. The most upregulated DEGs at 0 D versus those at 1 M and at 0 D versus those at 2 M in the litchi were system clustered into lipid metabolism processes, such as alpha-linolenic acid and linoleic acid metabolism (linoleate 13S-lipoxygenase 2-1; allene oxide cyclase 2; linoleate 13S-lipoxygenase 2-1; 4-coumarate–CoA ligase-like 5; linoleate 13S-lipoxygenase 2-1; TR107818| c4_g1, cytochrome P450 83A1; Supplementary Table 3 and **Figure 6**). Certain 2-hydroxy fatty acids comprise the putative categories of root exudate signals perceived by *Gigaspora* species; several genes related to fatty-acid and lipid metabolism that are highly upregulated in AM roots were identified (Gaude et al., 2012). Whether alpha-linolenic acid and linoleic acid could regulate AM colonization under decreased carbohydrate conditions in litchi mycorrhizal tree requires additional study. In addition to the signal molecules and defense-related genes in the symbiotic process, the DEGs involved in AM maintenance, such as transcription factors (**Figure 5A**), lectin and chitinase genes were also screened (**Figure 6A**; Supplementary Table 4). The functions of the transcription factors in the AP2, ERF, Myb, WRKY, and bHLH families as well as lectin genes were involved in cellular reprogramming during AM development (De Hoff et al., 2009; Hogekamp et al., 2011; Handa et al., 2015). This finding suggested that carbohydrate shortage potentially influence AM formation, maintenance, and systematic functioning from pre-symbiotic to post-symbiotic periods in litchi roots.

For AM fungal component, the E3 ubiquitin ligase complex SCF subunit scon-3 and putative ubiquitin thioesterase otu1 were identified in the upregulated DEGs at 0 D versus those at 1 W and at 0 D versus those at 2 W. The E3 ubiquitin ligase was found to ubiquitinate *Arabidopsis* receptor kinase flagellin sensing 2, which was also identified as agents interacting with symbiotic RLKs in *L. japonicus* (Robatzek et al., 2006; Mbengue et al., 2010; Den Herder et al., 2012). Both the genes encoding the E3 ubiquitin ligase complex SCF subunit scon-3 and putative ubiquitin thioesterase otu1 were upregulated in AM fungi upon recognition of carbohydrate decrease, which illustrated the ubiquitination was important in the process of AM fungi perceived carbohydrate decreased. The KEGG pathway of upregulated DEGs at 0 D versus those at 1 M and at 0 D versus those at 2 M in the AM fungi suggested that AM fungi not only relied on the variation of carbohydrate metabolism for the adaptation process but also upregulated the transcription in arginine and proline metabolism, alanine, aspartate, and glutamate metabolism and lipid metabolism for adopting carbohydrate starvation (Supplementary Table 3). The unigenes mapped to lipid metabolism and genes encoding chitin syntheses varied during fungal adaptation under carbohydrate starvation (**Figure 6**). The two categories of unigenes possibly related to lipochito-oligosaccharides, short-chain chitin oligomers, and thyroid, which may be correlated to the recognition of host plant and AM fungi (Bates et al., 2012; Kobae et al., 2014; Wewer et al., 2014). Interestingly, the transcripts of cytochrome b-245 and LRR were noted in the DEGs of AM fungi (Supplementary Table 3; **Figure 6**). *R. irregularis* possesses over 200 CYPs according to domain prediction using the InterPro database (Park et al., 2008; Moktali et al., 2012; Tisserant et al., 2013). This number is relatively large for CYPs of a fungal species. Previous studies showed that CYPs include heme–thiolate proteins, which are located in the endoplasmic reticulum and catalyze the oxidation of various organic compounds, such as lipids and sterols (Črešnar and Petrič, 2011). The members of the CYP51 family are well-conserved housekeeping genes that participate in the 14-demethylation of sterol precursors (van den Brink et al., 1998; Črešnar and Petrič, 2011). The CYP diversification may be related to various metabolic processes and possible fungal adaptation to the soil environment and host plant roots. In addition to CYPs, LRR transcripts were noted in the DEG database entries on AM fungi. Various surfaces of the leucine-rich repeat LRR ectodomain superstructure are utilized for interaction with the cognate ligand in both plant and animal receptors. *Arabidopsis* LRR receptor-like kinase FLS2 and rice receptor kinase-like protein Xa21 possess large ectodomains that comprise 28 LRRs and 23 LRRs, respectively (Song et al., 1995; Gómez-Gómez and Boller, 2000), and are directly involved in elicitor binding (Chinchilla et al., 2006; Lee et al., 2009). Because the LRRs form versatile binding domains for plant proteins were involved in the process of plant–microbe interaction, the LRRs in the AM fungi binding the secreted proteins from host plant needed further study.

## Conclusion

Girdling decreased the glucose, fructose, sucrose, quebrachitol, and even starch concentrations in the litchi mycorrhizal roots, which induced a decrease in AM colonization. In this study, we revealed the gene expression profiles of both host litchi and AM fungi during the decreased carbohydrate conditions by RNA-seq analysis. Both transcripts of litchi (54,100 unigenes) and AM fungi (33,120 unigenes) were identified in the period of decreased carbohydrates. The DEG analysis of transcriptomes identified potential novel unigenes of both host litchi and AM fungi. DEG analysis showed that flavonoids, alpha-linolenic acid, and linoleic acid were the main factors that regulated AM colonization in litchi. However, the flavonoids might play a role in the recognition of the stages of decreasing carbohydrate content, and alpha-linolenic acid or linoleic acid may affect AM formation under the process of adaptation. Litchi trees stimulated the expression of the defense-related genes (NADP-dependent malic enzyme gene, endochitinase PR4, basic form of pathogenesis-related protein 1, LRR receptor-like serine/threonine-protein kinase EFR, cyclic nucleotide-gated ion channel 1, lignin-forming anionic peroxidase, and peroxidase 72) and downregulated the symbiosis signal-transduction genes (heat-shock protein 82, heat-shock 70 kDa protein, putative heat-shock protein HSP 90-beta-3, transcription factor MYC4, probable protein phosphatase 2C 24, and regulatory protein NPR5) to inhibit new AM colonization. In addition to new AM colonization, carbohydrate shortage changed the transcription factor in the AP2, ERF, Myb, WRKY, bHLH families and lectin genes of litchi, which influenced AM maintenance in the post-symbiotic stage. Similar to those of the litchi host, the E3 ubiquitin ligase complex SCF subunit scon-3 and polyubiquitin of AM fungi were all upregulated at the perceived stages. This occurrence suggested that the ubiquitination process plays an important role in the AM fungal recognition of carbohydrate decrease. The transcription of cytochrome b-245 and LRRs were noted in the DEGs database, implying that these transcripts play important roles in the process of AM fungal adaptation under carbohydrate starvation despite the gene function still being largely unknown.

## Author Contributions

The data was interpreted and the article was drafted by BS. The manuscript was revised by SS. Meanwhile, the experiments were conceived and designed by BS and SS. BS, LL, and YW performed the experiments. BS and WL analyzed the data.

## Conflict of Interest Statement

The authors declare that the research was conducted in the absence of any commercial or financial relationships that could be construed as a potential conflict of interest.

## Footnotes

1 http://genome.jgi.doe.gov/Gloin1/Gloin1.home.html

2 http://www.ncbi.nlm.nih.gov

3 http://www.expasy.ch/sprot

4 http://www.genome.jp/kegg

5 http://www.uniprot.org/uniref/?query=&fil=identity:0.9

## Supplementary Material

FIGURE S1 **The girdling litchi trees in the experimental orchard. The girdling wound was deep to xylem with 0.5 cm width**.

FIGURE S2 **Flow chart of RNA-seq analysis**.

## Abbreviations

AM: arbuscular mycorrhiza
COG: cluster of orthologous groups of proteins
CYP: cytochrome P450
DEG: differentially expressed gene
FDR: false discovery rate
FPKM: fragments per kilobase of exon per million fragments mapped
Gloin1: *Rhizophagus irregularis* genome assembly
GO: gene ontology
HPLC: high-performance liquid chromatography
KEGG: Kyoto Encyclopedia of Genes and Genomes
LRR: leucine-rich repeats
nr: NCBI non-redundant (nr) database.

## References

[B1] Baier M. C. Keck M. Gödde V. Niehaus K. Küster H. Hohnjec N. (2010). Knockdown of the symbiotic sucrose synthase MtSucS1 affects arbuscule maturation and maintenance in mycorrhizal roots of *Medicago truncatula*. *Plant Physiol.* 152 1000–1014. 10.1104/pp.109.149898 20007443 PMC2815868

[B2] Bates P. D. Fatihi A. Snapp A. R. Carlsson A. S. Browse J. Lu C. (2012). Acyl editing and headgroup exchange are the major mechanisms that direct polyunsaturated fatty acid flux into triacylglycerols. *Plant Physiol.* 160 1530–1539. 10.1104/pp.112.204438 22932756 PMC3490606

[B3] Bécard G. Douds D. D. Pfeffer P. E. (1992). Extensive in vitro hyphal growth of vesicular-arbuscular mycorrhizal fungi in the presence of CO_2_ and flavonols. *Appl. Environ. Microbiol.* 58 821–825. 16348673 10.1128/aem.58.3.821-825.1992 PMC195340

[B4] Bécard G. Taylor L. P. Douds D. D. Pfeffer P. E. Doner L. W. (1995). Flavonoids are not necessary plant signal compounds in arbuscular mycorrhizal symbioses. *Mol. Plant Microbe Ineract.* 8 252–258. 10.1094/mpmi-8-0252

[B5] Besserer A. Bécard G. Jauneau A. Roux C. Séjalon-Delmas N. (2008). GR24, a synthetic analog of strigolactones, stimulates the mitosis and growth of the arbuscular mycorrhizal fungus *Gigaspora rosea* by boosting its energy metabolism. *Plant Physiol.* 148 402–413. 10.1104/pp.108.121400 18614712 PMC2528133

[B6] Bonneau L. Huguet S. Wipf D. Pauly N. Truong H. N. (2013). Combined phosphate and nitrogen limitation generates a nutrient stress transcriptome favorable for arbuscular mycorrhizal symbiosis in *Medicago truncatula*. *New Phytol.* 199 188–202. 10.1111/nph.12234 23506613

[B7] Bucher M. Hause B. Krajinski F. Küster H. (2014). Through the doors of perception to function in arbuscular mycorrhizal symbioses. *New Phytol.* 204 833–840. 10.1111/nph.12862 25414918

[B8] Chinchilla D. Bauer Z. Regenass M. Boller T. Felix G. (2006). The *Arabidopsis* receptor kinase FLS2 binds flg22 and determines the specificity of flagellin perception. *Plant Cell* 18 465–476. 10.1105/tpc.105.036574 16377758 PMC1356552

[B9] Črešnar B. Petrič S. (2011). Cytochrome P450 enzymes in the fungal kingdom. *BBA-Proteins Proteom.* 1814 29–35. 10.1016/j.bbapap.2010.06.020 20619366

[B10] De Hoff P. L. Brill L. M. Hirsch A. M. (2009). Plant lectins: the ties that bind in root symbiosis and plant defense. *Mol. Genet. Genom.* 282 1–15. 10.1007/s00438-009-0460-8 PMC2695554 19488786

[B11] Den Herder G. Yoshida S. Antolín-Llovera M. Ried M. K. Parniske M. (2012). *Lotus japonicus* E3 ligase SEVEN IN ABSENTIA4 destabilizes the symbiosis receptor-like kinase SYMRK and negatively regulates rhizobial infection. *Plant Cell* 24 1691–1707. 10.1105/tpc.110.082248 22534128 PMC3398572

[B12] Fellbaum C. R. Gachomo E. W. Beesetty Y. Choudharib S. Strahanc G. D. Pfefferc P. E. (2012). Carbon availability triggers fungal nitrogen uptake and transport in arbuscular mycorrhizal symbiosis. *Proc. Natl. Acad. Sci. U.S.A.* 109 2666–2671. 10.1073/pnas.1118650109 22308426 PMC3289346

[B13] Fiorilli V. Vallino M. Biselli C. Faccio A. Bagnaresi P. Bonfante P. (2015). Host and non-host roots in rice: cellular and molecular approaches reveal differential responses to arbuscular mycorrhizal fungi. *Front. Plant Sci.* 6:636 10.3389/fpls.2015.00636 PMC4534827 26322072

[B14] Gallou A. Declerck S. Cranenbrouck S. (2012). Transcriptional regulation of defence genes and involvement of the WRKY transcription factor in arbuscular mycorrhizal potato root colonization. *Funct. Integr. Genomic* 12 183–198. 10.1007/s10142-011-0241-4 21811781

[B15] Garber M. Grabherr M. G. Guttman M. Trapnell C. (2011). Computational methods for transcriptome annotation and quantification using RNA-seq. *Nat. Methods* 8 469–477. 10.1038/nmeth.1613 21623353

[B16] Gaude N. Bortfeld S. Duensing N. Lohse M. Krajinski F. (2012). Arbuscule-containing and non-colonized cortical cells of mycorrhizal roots undergo a massive and specific reprogramming during arbuscular mycorrhizal development. *Plant J.* 69 510–528. 10.1111/j.1365-313x.2011.04810.x 21978245

[B17] Genre A. Chabaud M. Balzergue C. Puech-Pagès V. Novero M. Rey T. (2013). Short-chain chitin oligomers from arbuscular mycorrhizal fungi trigger nuclear Ca2+ spiking in *Medicago truncatula* roots and their production is enhanced by strigolactone. *New Phytol.* 198 190–202. 10.1111/nph.12146 23384011

[B18] Gómez-Gómez L. Boller T. (2000). FLS2: an LRR receptor–like kinase involved in the perception of the bacterial elicitor flagellin in *Arabidopsis*. *Mol. Cell.* 5 1003–1011. 10.1016/S1097-2765(00)80265-8 10911994

[B19] Grabherr M. G. Haas B. J. Yassour M. Levin J. Z. Thompson D. A. Amit I. (2011). Full-length transcriptome assembly from RNA-Seq data without a reference genome. *Nat. Biotechnol.* 29 644–652. 10.1038/nbt.1883 21572440 PMC3571712

[B20] Handa Y. Nishide H. Takeda N. Suzuki Y. Kawaguchi M. Saito K. (2015). RNA-seq transcriptional profiling of an arbuscular mycorrhiza provides insights into regulated and coordinated gene expression in *Lotus japonicus* and *Rhizophagus irregularis*. *Plant Cell Physiol.* 56 1490–1511. 10.1093/pcp/pcv071 26009592

[B21] Hayman D. S. (1974). Plant growth responses to vesicular-arbuscular mycorrhiza.VI. Effect of light and temperature. *New Phytol.* 73 71–80.

[B22] Helber N. Wippel K. Sauer N. Schaarschmidt S. Hause B. Requena N. (2011). A versatile monosaccharide transporter that operates in the arbuscular mycorrhizal fungus *Glomus* sp is crucial for the symbiotic relationship with plants. *Plant Cell* 23 3812–3823. 10.1105/tpc.111.089813 21972259 PMC3229151

[B23] Hogekamp C. Arndt D. Pereira P. Becker J. D. Hohnjec N. Küster H. (2011). Laser microdissection unravels cell-type-specific transcription in arbuscular mycorrhizal roots, including CAAT-Box transcription factor gene expression correlating with fungal contact and spread. *Plant Physiol.* 157 2023–2043. 10.1104/pp.111.186635 22034628 PMC3327204

[B24] Hogekamp C. Küster H. (2013). A roadmap of cell-type specific gene expression during sequential stages of the arbuscular mycorrhiza symbiosis. *BMC Genomics* 14:306 10.1186/1471-2164-14-306 PMC3667144 23647797

[B25] Huang X. M. Wang H. C. Yuan W. Q. (2003). Effects of twig girdling at different stages on new shoot growth and carbon nutrient reservation. *Acta Hortic. Sin.* 30 192–194.

[B26] Kiers E. T. Duhamel M. Beesetty Y. Mensah J. A. Franken O. Verbruggen E. (2011). Reciprocal rewards stabilize cooperation in the mycorrhizal symbiosis. *Science* 333 880–882. 10.1126/science.1208473 21836016

[B27] Kobae Y. Gutjahr C. Paszkowski U. Kojima T. Fujiwara T. Hata S. (2014). Lipid droplets of arbuscular mycorrhizal fungi emerge in concert with arbuscule collapse. *Plant Cell Physiol.* 55 1945–1953. 10.1093/pcp/pcu123 25231957

[B28] Lee S. W. Han S. W. Sririyanum M. Park C. J. Seo Y. S. Ronald P. C. (2009). A type I–secreted, sulfated peptide triggers XA21-mediated innate immunity. *Science* 326 850–853. 10.1126/science.1173438 19892983

[B29] Li B. Dewey C. N. (2011). RSEM: accurate transcript quantification from RNA-Seq data with or without a reference genome. *BMC Bioinformatics* 12:323 10.1186/1471-2105-12-323 PMC3163565 21816040

[B30] Maillet F. Poinsot V. André O. Puech-Pagès V. Haouy A. Gueunier M. (2011). Fungal lipochitooligosaccharide symbiotic signals in arbuscular mycorrhiza. *Nature* 469 58–63. 10.1038/nature09622 21209659

[B31] Mbengue M. Camut S. de Carvalho-Niebel F. Deslandes L. Froidure S. Klaus-Heisen D. (2010). The *Medicago truncatula* E3 ubiquitin ligase PUB1 interacts with the LYK3 symbiotic receptor and negatively regulates infection and nodulation. *Plant Cell* 22 3474–3488. 10.1105/tpc.110.075861 20971894 PMC2990133

[B32] McGonigle T. P. Miller M. H. Evans D. G. Fairchild G. L. Swan J. A. (1990). A new method which gives an objective measure of colonization of roots by vesicular-arbuscular mycorrhizal fungi. *New Phytol.* 115 495–501. 10.1111/j.1469-8137.1990.tb00476.x 33874272

[B33] Miyata K. Kozaki T. Kouzai Y. Ozawa K. Ishii K. Asamizu E. (2014). The bifunctional plant receptor, OsCERK1, regulates both chitin-triggered immunity and arbuscular mycorrhizal symbiosis in rice. *Plant Cell Physiol.* 55 1864–1872. 10.1093/pcp/pcu129 25231970

[B34] Moktali V. Park J. Fedorova-Abrams N. Park B. Choi J. Lee Y. H. (2012). Systematic and searchable classification of cytochrome P450 proteins encoded by fungal and oomycete genomes. *BMC Genomics* 13:525 10.1186/1471-2164-13-525 PMC3505482 23033934

[B35] Nagahashi G. Douds D. D. (2011). The effects of hydroxy fatty acids on the hyphal branching of germinated spores of AM fungi. *Fungal Boil.* 115 351–358. 10.1016/j.funbio.2011.01.006 21530917

[B36] Park J. Lee S. Choi J. Ahn K. Park B. Park J. (2008). Fungal cytochrome P450 database. *BMC Genomics* 9:402 10.1186/1471-2164-9-402 PMC2542383 18755027

[B37] Parniske M. (2008). Arbuscular mycorrhiza: the mother of plant root endosymbioses. *Nat. Rev. Microbiol.* 6 763–775. 10.1038/nrmicro1987 18794914

[B38] Phillips J. M. Hayman D. S. (1970). Improved procedures for clearing roots and staining parasitic and vesicular-arbuscular mycorrhizal fungi for rapid assessment of infection. *Trans. Br. Mycol. Soc.* 55 158–161. 10.1016/s0007-1536(70)80110-3

[B39] Robatzek S. Chinchilla D. Boller T. (2006). Ligand-induced endocytosis of the pattern recognition receptor FLS2 in *Arabidopsis*. *Gene Dev.* 20 537–542. 10.1101/gad.366506 16510871 PMC1410809

[B40] Ruzicka D. R. Chamala S. Barriosmasias F. H. Martin F. Smith S. Jackson L. E. (2013). Inside arbuscular mycorrhizal roots – molecular probes to understand the symbiosis. *Plant Genome* 6 494–494. 10.3835/plantgenome2012.06.0007

[B41] Ruzicka D. R. Hausmann N. T. Barrios-Masias F. H. Jackson L. E. Schachtman D. P. (2012). Transcriptomic and metabolic responses of mycorrhizal roots to nitrogen patches under field conditions. *Plant Soil* 350 145–162. 10.1007/S11104-011-0890-Z

[B42] Schaarschmidt S. González M. C. Roitsch T. Strack D. Sonnewald U. Hause B. (2007). Regulation of arbuscular mycorrhization by carbon. The symbiotic interaction cannot be improved by increased carbon availability accomplished by root-specifically enhanced invertase activity. *Plant Physiol.* 143 1827–1840. 10.1104/pp.107.096446 17416641 PMC1851815

[B43] Smith S. E. Read D. J. (2008). *Mycorrhizal Symbiosis*, 3nd Edn. New York, NY: Academic Press 10.1016/B978-012370526-6.50003-9

[B44] Song W. Y. Wang G. L. Chen L. L. Kim H. S. Pi L. Y. Holsten T. (1995). A receptor kinase-like protein encoded by the rice disease resistance gene, XA21. *Science* 270 1804–1806. 10.1126/science.270.5243.1804 8525370

[B45] Sun J. Miller J. B. Granqvist E. Wiley-Kalil A. Gobbato E. Maillet F. (2015). Activation of symbiosis signaling by arbuscular mycorrhizal fungi in legumes and rice. *Plant Cell* 27 828–838. 10.1105/tpc.114.131326 PMC4558648 25724637

[B46] Tisserant E. Malbreil M. Kuo A. Kohler A. Symeonidi A. Balestrini R. (2013). Genome of an arbuscular mycorrhizal fungus provides insight into the oldest plant symbiosis. *Proc. Natl. Acad. Sci. U.S.A.* 110 20117–20122. 10.1073/pnas.1313452110 24277808 PMC3864322

[B47] Tromas A. Parizot B. Diagne N. Champion A. Hocher V. Cissoko M. (2012). Heart of endosymbioses: transcriptomics reveals a conservedgenetic program among arbuscular mycorrhizal, actinorhizal and legume-rhizobial symbioses. *PLoS ONE* 7:e44742 10.1371/journal.pone.0044742 PMC3435296 22970303

[B48] Tromp J. (1983). Nutrient reserves in roots of fruit trees, in particular carbohydrates and nitrogen. *Plant Soil.* 71 401–413 10.1007/BF02182682

[B49] van den Brink H. M. van Gorcom R. F. M. van den Hondel C. A. M. J. J. Punt P. J. (1998). Cytochrome P450 enzyme systems in fungi. *Fungal Genet. Biol.* 23 1–17. 10.1006/fgbi.1997.1021 9501474

[B50] Vierheilig H. Bago B. Lerat S. Piché Y. (2002). Shoot-produced, light dependent factors are partially involved in the expression of the arbuscular mycorrhizal (AM) status of AM host and non-host plants. *J. Plant Nutr. Soil Sci.* 165 21–25. 10.1002/1522-2624(200202)165:1<21::AID-JPLN21>3.0.CO;2-9

[B51] Wang H. C. Wu Z. H. Huang X. M. Hu G. B. Chen H. B. (2013). Determination of quebrachitol *Litchi chinensis* and *Dimocarpus longan* in sapindacea family. *J. S. China Agric. Univ.* 34 315–319.

[B52] Wang C. Gong B. Bushel P. R. Thierry-Mieg J. Thierry-Mieg D. Xu J. (2014). The concordance between RNA-seq and microarray data depends on chemical treatment and transcript abundance. *Nat. Biotechnol.* 32 926–932. 10.1038/nbt.3001 25150839 PMC4243706

[B53] Wang T. D. Zhang H. F. Wu Z. C. Li J. G. Huang X. M. Wang H. C. (2014). Sugar uptake in the aril of litchi fruit depends on the apoplasmic post-phloem transport and the activity of proton pumps and the putative transporter LcSUT4. *Plant Cell Physiol.* 56 377–387. 10.1093/pcp/pcu173 25432972

[B54] Wewer V. Brands M. Dörmann P. (2014). Fatty acid synthesis and lipid metabolism in the obligate biotrophic fungus *Rhizophagus irregularis* during mycorrhization of *Lotus japonicus*. *Plant J.* 79 398–412. 10.1038/nbt.3001 24888347

[B55] Xie C. Mao X. Huang J. Ding Y. Wu J. Dong S. (2011). KOBAS 2.0: a web server for annotation and identification of enriched pathways and diseases. *Nucleic Acids Res.* 39 W316–W322. 10.1093/nar/gkr483 21715386 PMC3125809

[B56] Young M. D. Wakefield M. J. Smyth G. K. Oshlack A. (2010). Method Gene Ontology Analysis for RNA-Seq: accounting for selection bias. *Genome Biol.* 11:R14 10.1186/gb-2010-11-2-r14 PMC2872874 20132535

[B57] Yuan R. C. Huang H. B. (1993). Regulation of roots and shoots growth and fruit-drop of young litchi trees by trunk girdling in view of source-sink relationships. *J. Fruit Sci.* 10 195–198.

