# Supplementary figures and images for: Transcriptomes of Arbuscular Mycorrhizal Fungi and Litchi Host Interaction after Tree Girdling
Source: Front Microbiol. 2016 Mar 30;7:408. doi: 10.3389/fmicb.2016.00408 (PMC4811939; doi:10.3389/fmicb.2016.00408)

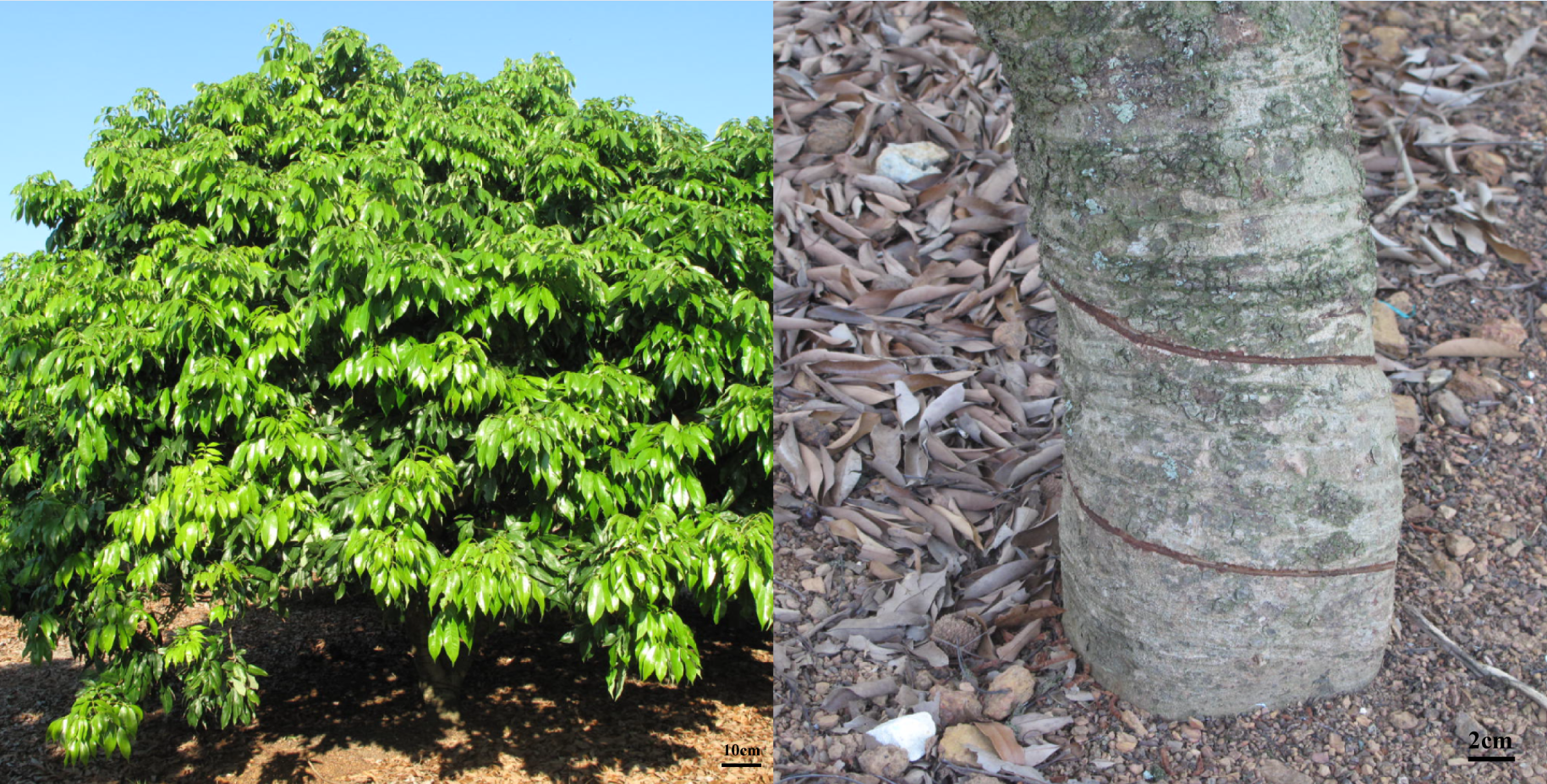

Supplement: FIGURE S1 — The girdling litchi trees in the experimental orchard. The girdling wound was deep to xylem with 0.5 cm width. [file Image_1.TIF]

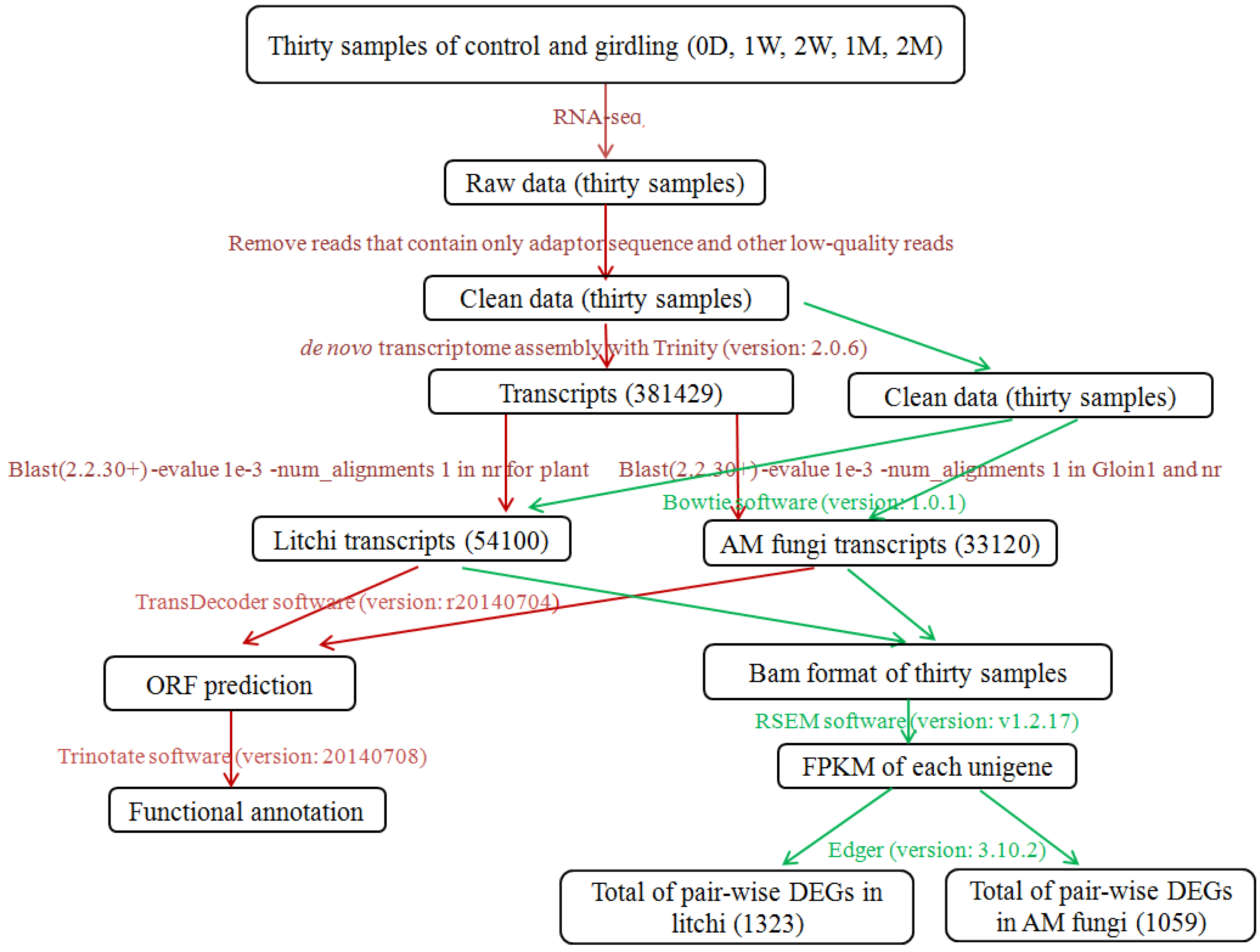

Supplement: FIGURE S2 — Flow chart of RNA-seq analysis. [file Image_2.TIF]
