# Supplementary material for: Transcriptomes of Arbuscular Mycorrhizal Fungi and Litchi Host Interaction after Tree Girdling
Source: Front Microbiol. 2016 Mar 30;7:408. doi: 10.3389/fmicb.2016.00408 (PMC4811939; doi:10.3389/fmicb.2016.00408)
Supplement: Supplementary file 3 [file Table_1.DOCX]

Supplemental Table 1 The pH value, organic matter and mineral elements in litchi [orchard](javascript:showjdsw('showjd_0','j_0')).

|  |  | pH | organic matter（%） | [cation exchange capacity](javascript:showjdsw('showjd_0','j_0'))（mg/kg） | available N（mg/kg） | available P（mg/kg） | available K（mg/kg） |
| --- | --- | --- | --- | --- | --- | --- | --- |
| 0D | control | 4.8 ± 0.17 | 2.2 ± 0.29 | 12.6 ± 1.5 | 120 ± 15.6 | 19 ± 3.1 | 222 ± 29.5 |
|  | girdling | 4.9 ± 0.15 | 2.3 ± 0.31 | 13.5 ± 1.2 | 117 ± 15.6 | 23 ± 4.3 | 216 ± 17.3 |
| 2M | control | 4.9 ± 0.62 | 2.2 ± 0.29 | 11.9 ± 1.6 | 115 ± 15.64 | 19 ± 3.1 | 200 ± 29.5 |
|  | girdling | 4.9 ± 0.25 | 2.3 ± 0.57 | 11.6 ± 1.8 | 108 ± 10.6 | 17 ± 2.3 | 195 ± 23 |

|  |  | available Ca（mg/kg） | available Mg（mg/kg） | available S（mg/kg） | available Cu（mg/kg） | available Zn（mg/kg） | available B（mg/kg） | available Mo（mg/kg） |
| --- | --- | --- | --- | --- | --- | --- | --- | --- |
| 0D | control | 407 ± 50.3 | 73.5 ± 10.6 | 85.3 ± 13.07 | 3.35 ± 0.85 | 3.08 ± 0.97 | 0.73 ± 0.08 | 0.19 ± 0.03 |
|  | girdling | 412 ± 42.3 | 78.9 ± 10.3 | 79.5 ± 8.05 | 3.58 ± 0.78 | 2.88 ± 0.62 | 0.84 ± 0.11 | 0.18 ± 0.04 |
| 2M | control | 418 ± 20.8 | 70.9 ± 10.1 | 85.3 ± 13.02 | 3.35 ± 0.83 | 3.08 ± 0.99 | 0.73 ± 0.08 | 0.19 ± 0.03 |
|  | girdling | 398 ± 30.3 | 68.9 ± 10.2 | 77.5 ± 8.07 | 3.18 ± 0.34 | 2.98 ± 0.64 | 0.75 ± 0.15 | 0.15 ± 0.06 |
