# Supplementary material for: Transcriptomes of Arbuscular Mycorrhizal Fungi and Litchi Host Interaction after Tree Girdling
Source: Front Microbiol. 2016 Mar 30;7:408. doi: 10.3389/fmicb.2016.00408 (PMC4811939; doi:10.3389/fmicb.2016.00408)
Supplement: Supplementary file 4 [file Table_2.DOCX]

Supplemental Table 2 Summary of read numbers based on the RNA-Seq data from the each mycorrhizal roots sample of litchi after girdling treatment.

| **Sample name** | **Total reads** | **Total bases** | **GC content** | **Q20** | **Q30** |
| --- | --- | --- | --- | --- | --- |
| C0D_1 | 62,346,818 | 6,234,681,800 | 44.84% | 99.02% | 94.64% |
| C0D_2 | 62,300,630 | 6,230,063,000 | 45.00% | 99.02% | 94.65% |
| C0D_3 | 60,469,284 | 6,046,928,400 | 44.59% | 99.03% | 94.72% |
| C1W_1 | 67,977,948 | 6,797,794,800 | 44.36% | 97.16% | 90.65% |
| C1W_2 | 59,595,370 | 5,959,537,000 | 44.75% | 97.11% | 90.52% |
| C1W_3 | 64,194,728 | 6,419,472,800 | 44.47% | 97.14% | 90.61% |
| C2W_1 | 67,762,938 | 6,776,293,800 | 44.47% | 97.27% | 90.91% |
| C2W_2 | 61,195,068 | 6,119,506,800 | 44.58% | 97.23% | 90.80% |
| C2W_3 | 69,213,094 | 6,921,309,400 | 43.95% | 97.26% | 90.89% |
| C1M_1 | 64,079,844 | 6,407,984,400 | 46.06% | 97.19% | 90.55% |
| C1M_2 | 61,556,354 | 6,155,635,400 | 44.90% | 97.30% | 90.87% |
| C1M_3 | 64,505,376 | 6,450,537,600 | 44.39% | 97.39% | 91.13% |
| C2M_1 | 66,080,550 | 6,608,055,000 | 44.68% | 96.99% | 90.02% |
| C2M_2 | 63,113,656 | 6,311,365,600 | 44.35% | 96.87% | 89.66% |
| C2M_3 | 62,449,872 | 6,244,987,200 | 44.98% | 96.79% | 89.42% |
| G0D_1 | 62,293,452 | 6,229,345,200 | 45.04% | 99.03% | 94.71% |
| G0D_2 | 62,216,000 | 6,221,600,000 | 45.26% | 99.02% | 94.62% |
| G0D_3 | 60,541,046 | 6,054,104,600 | 44.55% | 99.04% | 94.74% |
| G1W_1 | 57,906,038 | 5,790,603,800 | 44.68% | 97.17% | 90.67% |
| G1W_2 | 64,487,922 | 6,448,792,200 | 44.62% | 97.21% | 90.81% |
| G1W_3 | 60,880,060 | 6,088,006,000 | 44.76% | 97.20% | 90.76% |
| G2W_1 | 58,102,690 | 5,810,269,000 | 45.02% | 97.22% | 90.76% |
| G2W_2 | 63,842,054 | 6,384,205,400 | 45.20% | 97.15% | 90.54% |
| G2W_3 | 60,893,572 | 6,089,357,200 | 44.92% | 97.20% | 90.70% |
| G1M_1 | 61,200,116 | 6,120,011,600 | 44.49% | 97.28% | 90.83% |
| G1M_2 | 65,328,636 | 6,532,863,600 | 45.66% | 97.25% | 90.73% |
| G1M_3 | 63,184,358 | 6,318,435,800 | 44.15% | 97.37% | 91.10% |
| G2M_1 | 61,790,124 | 6,179,012,400 | 44.05% | 97.05% | 90.20% |
| G2M_2 | 61,534,878 | 6,153,487,800 | 44.25% | 96.99% | 90.01% |
| G2M_3 | 61,396,992 | 6,139,699,200 | 45.68% | 96.76% | 89.42% |

The C represents control, and the G represents girdling; 0D, 1W, 2W, 1M, 2M represent the time after treatment (0 day, 1 week, 2 weeks, 1 month and 2 months); 1, 2, 3 represents biological repeats.
